# Rapid transformation of two libraries using Kotter’s Eight Steps of Change

**DOI:** 10.5195/jmla.2017.97

**Published:** 2017-07-01

**Authors:** Terrie R. Wheeler, Kristi L. Holmes

## Abstract

**Background:**

Two new directors were each charged by their institutions to catalyze transformational change in their libraries and to develop dynamic and evolving information ecosystems ready for the information challenges of the future. The directors approached this transformational change using a strategic, forward-looking approach.

**Results:**

This paper presents examples of actions that served as catalysts for change at the two libraries using Kotter’s Eight Steps of Change as a framework. Small and large changes are critical for successfully transforming library services, resources, and personnel.

**Conclusions:**

Libraries are faced with incredible pressure to adapt to meet emerging and intensifying information needs on today’s academic medical campuses. These pressures offer an opportunity for libraries to accelerate their evolution at the micro and macro levels. This commentary reports the expansion of new services and areas of support, enhancement of professional visibility of the libraries on their campuses, and overall, a more positive and productive environment at the respective institutions.

## INTRODUCTION

**Figure 1:**
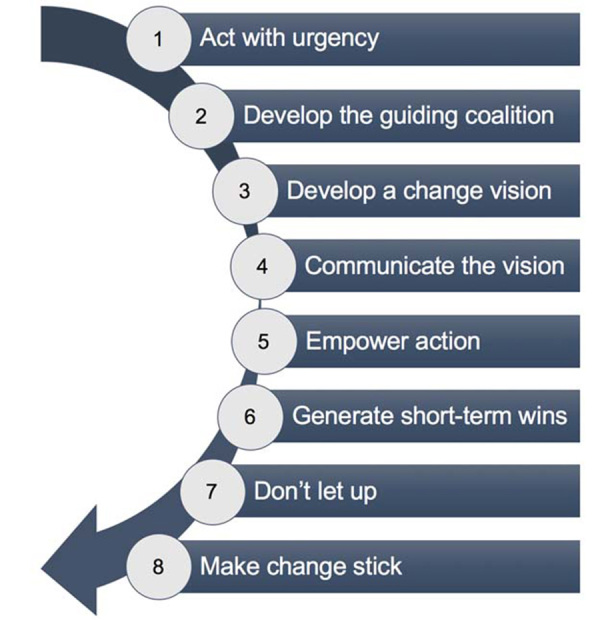
Kotter’s Eight Steps of Change

Academic libraries are faced with incredible pressure to adapt to successfully meet emerging and intensifying information needs on campus. Two new health sciences library directors were each charged by their institutions to catalyze transformational change in their libraries and to develop dynamic and evolving information ecosystems ready for the information challenges of the future. The directors approached this process using a strategic, forward-looking approach that can be mapped onto Kotter’s Eight Steps of Change [1] (Figure 1). This step-wise approach enabled the expansion of new services and areas of support, enhanced the professional visibility of the libraries on their campuses, and helped create a more positive and productive environment at their respective institutions.

## STEP 1: ACT WITH URGENCY

A sense of urgency is created by an outside event, perception, or change that triggers new approaches to library services. Kotter calls this a sense of urgency because the change can be perceived as a threat to familiar approaches, but a leader can help enable the group to try promising new methods.

At the Northwestern University Feinberg School of Medicine’s Galter Health Sciences Library, the sense of urgency was acutely demonstrated by a change in the reporting structure of the library to the Northwestern University Clinical and Translational Sciences Institute (NUCATS) to capitalize on synergies across these two units [2]. At Weill Cornell Medicine’s Samuel J. Wood Library, the sense of urgency was shown in the provost’s new focus on research, which led to a greater demand for new research library services and library efforts to secure grant funding. In both cases, the library discovered opportunities to demonstrate its value by developing innovative library services to support the research enterprise.

Acting with urgency should first be grounded in a clear understanding of organizational needs. The new directors spent their first several months actively listening to their teams, leadership, and diverse stakeholders to determine organizational needs and best practices for accomplishing meaningful change. Active listening enabled improved communication among the libraries, campus communities, and senior organizational leadership. The shared sense of urgency with campus leadership ultimately helped drive the actions required to implement change.

## STEP 2: DEVELOP THE GUIDING COALITION

Leading change is rarely done single-handedly. Each director identified existing and new key staff members who shared the urgency and who were invested in developing innovative new solutions. These individuals were willing to learn new skills, embraced change, actively listened, contributed ideas, and were trusted by others. These attributes were essential to enable them to model change in the organization. Each director encouraged this guiding coalition to work as a team by first contributing to the team effort themselves. Kouzes and Posner note that leaders are most effective when they live shared values and teach others to model those same values [3]. Both directors worked to model teamwork and lauded it when they saw it, because the work necessary to achieve the vision was too difficult to accomplish by only a few.

At the Wood Library, the director consciously made efforts to win the hearts of the staff through communicating clearly and setting an example [4]. A weekly message to all library staff highlighted peer examples of teamwork and became known as “This Week at Wood.” Ad hoc teams of internal experts called “tiger teams” were formed to make quick progress, and the director participated on many of these teams to provide encouragement and leadership. At the Galter Library, a workgroup structure was implemented, taking the real work of the library out of the formal organizational chart and into agile teams to more effectively and efficiently accomplish the work. These cross-library workgroups have ensured that all voices are heard and that diverse perspectives are considered.

## STEP 3: DEVELOP A CHANGE VISION

Each director presented a vision of change to university leadership before being hired. Once at work, each director conducted active listening sessions with university leaders and library staff. Thus, each could create a vision that met the needs of the organization while capitalizing on the strengths of the library team. Two years later, each director has enacted much of what she envisioned and shared during the interview process.

At each site, the change vision was a radical departure from what was previously recognized as library activity. Each library developed a range of services that were heavily focused around research and burgeoning clinical support services, while enhancing efforts to support the education mission of the institutions. Both directors also adapted strategies to achieve the vision, including hiring team members with new skill sets to deliver innovative services. As with many other libraries, both sites departed from physical collections and instead capitalized on electronic content. An added by-product of the new vision was an increase in scholarly activity such as publications and grants by library team members.

## STEP 4: COMMUNICATE THE VISION

Each library director established key approaches to clearly communicate the vision. At the Galter Library, the vision forward was communicated regularly through a combined strategy of internal and external communications about topics such as new services and resources, library scholarship, introduction of new hires, and examples of excellence across the entire team. Early all-team emails morphed into regular communications via workgroups and team meetings. Outward-facing communications have been a critical component of the Galter Library’s overall communication strategy and include NUCATS communications [5, 6], a regular column in the Office of Research’s Breakthroughs newsletter [7], and a reinvigorated liaison program [8].

At the Wood Library, “This Week at Wood” messages highlighted library staff who demonstrated the core values of teamwork, excellence, innovation, or service that furthered library change. New services such as grant editing, curating a clinical data core, and developing automated bibliometric services were launched. To develop an explicit expression of the library’s core values and as a team-building exercise, the entire library staff convened to develop the Wood Library’s balanced scorecard and strategy map, which communicates the library’s vision to library staff and stakeholders. This adoption of core values and communication of the vision accelerated change.

## STEP 5: EMPOWER BROAD-BASED ACTION

Both directors encouraged their teams to make this process their own, encouraging thoughtful risk-taking and contribution of nontraditional ideas and actions to catalyze change. Likewise, both directors leveraged renovation plans to envision new spaces to support new library initiatives and promote collaboration.

Galter Library accelerated several key areas of support for the Feinberg School of Medicine, including development of a forward-looking digital architecture to support scholarly dissemination, preservation, and publications tracking. A Metrics and Impact Core (MIC) [9] was launched, increased emphasis was placed on library efforts toward big data, and the library’s successful systematic review service tripled. A next-generation scholarly repository offering machine-readable metadata was launched to promote new models of dissemination [10], and the Digital Initiatives work group implemented Symplectic Elements to collect, analyze, and showcase the outputs of academic research [11]. Renewed emphasis was placed on the Galter Library Special Collections unit, with major inventory and housekeeping projects to help ensure that the special collections unit is successful in its mission to preserve the heritage of the school. Finally, the Galter Library team looked for opportunities to foster dialog in critical areas such as diversity and ethics through the National Library of Medicine exhibition program [12] and partnerships with the Office of Diversity and Inclusion and the Office of Medical Education. These strategic actions were based on an environmental scan of existing and expected requirements related to federally funded research, analysis of the literature for research trends and trajectories, scrutiny of existing areas of excellence, and institutional priorities.

At the Wood Library, several broad-based nontraditional actions empowered library faculty and staff to develop events and services that they had never imagined before. These included a clinical librarian program that has more than doubled in staff size to meet an exponentially growing demand for clinical services, including an internship program with a full year of curriculum. The team at Weill Cornell also emphasized scholarly publications to champion clinical systematic reviews and prepare case reports for the literature, building on an already successful systematic reviews service [13]. One exciting opportunity for action toward achieving the library’s vision for excellence, innovation, teamwork, and customer service is the now-annual SMARTFest technology fair [14], which grew from a small experiment to a campus-wide affair drawing over 1,100 participants over the 3-hour event. The success of SMARTFest was recognized by the provost and the vice-provost during their executive meetings, bringing more attention to the library as a partner for large-scale initiatives. These accomplishments not only changed leadership’s perception of the library, but also changed library staff’s perception of themselves and their capabilities.

## STEP 6: GENERATE SHORT-TERM WINS

Short-term wins are highly visible changes that quickly propel change forward. Many examples from both libraries—such as massive collection weeding projects, renovations, and development of new events and services—all have highly visible elements. Both libraries have been successful in generating short-term wins by delivering highly visible results of organization-wide importance in a timely manner, including support for research proposals through letters of support and boilerplate language about library resources and services. Some critical advancements might not be as visible, however. It is imperative to call attention to these less visible instances by recognizing efforts so that they are visible to the rest of the team and the larger organization.

The Galter Library director also serves as the director of evaluation for NUCATS and for other center and training grants, including the Chicago Cancer Health Equity Collaborative [15] funded by the National Cancer Institute to three Chicago-area institutions. Collaborating on proposals and identifying early wins on these projects enables her to highlight potential opportunities for collaboration between the projects and the library team and funded efforts to support the growth and visibility of Galter Library in supporting strategic areas at the Feinberg School of Medicine.

The Wood Library director wrote the library’s first successful National Library of Medicine informationist supplement [16] and collaborated on the recent Clinical and Translational Science Award (CTSA) proposal. In partnership with the CTSA institute and the Department of Healthcare Policy and Research, the Wood Library has developed a data curation service for a Health Insurance Portability and Accountability Act (HIPAA)–compliant data core for health data analysis. Wood Library also developed specifications for an academic staff management system to provide data management of the faculty affairs workflow, including appointment, tenure, promotion, annual review, and publications management activities.

In each case, the library directors worked to highlight the efforts of library staff so that these short-term wins were visible to a larger audience, and the visibility of the short-term wins helped the libraries’ stature grow significantly. Making efforts to change visible also helps employees realize that they are a critical part of the process. To this end, a *Harvard Business Review* article notes that employees are motivated by progress more than they are motivated by money or prestige [17]. Highlighting short-term wins demonstrates progress and gets the entire team on board.

## STEP 7: DON’T LET UP

The increased visibility created by the short-term wins facilitates the next step: transforming the culture by changing policies or systems that oppose the vision. Both directors challenged traditional library roles and organizational structure by working with human resources to promote and develop opportunities for employees.

At the Wood Library, paraprofessional position descriptions were rewritten to support identity management activities [18] and launch a research grant–editing pilot program championed by campus leadership that has already brought over two million dollars to Weill Cornell in eight months [19]. Other positions were amended to add new services, such as curation of the data core. Galter Library considered the skillset needed to support the evolving library and developed an entirely new paraprofessional job family to support traditional library functions as well as contribute to new programs in research services, digital systems, and collection services.

Each library recognized the value of keeping the momentum going through this period of intense continuous improvement. As the libraries developed momentum and experienced progress, their teams were ready for more. Positive change brings opportunity to the transformed library. Both directors have strategically placed their libraries at the nexus of new informatics initiatives and expect critical roles in precision medicine data.

With the support of Weill Cornell leadership, the Wood Library is launching a bioinformatics support program and a master data management initiative for improved institutional reporting. The Galter Library is enhancing informatics and big data activities on campus, including efforts to support the Northwestern Medicine Enterprise Data Warehouse [20].

## STEP 8: MAKE CHANGE STICK

Many employees will grasp the connection between the new vision, the new behaviors, and their own newfound success. It is critical that this connection is not taken for granted! Both library directors work to regularly communicate how the new vision and choices have made the library successful. It is also essential to invest in leadership development and succession. Both library directors have established post-doctoral and internship or practicum programs. They also mentor their direct reports and encourage their staff to take chances so that staff can learn and be invested in solutions. The directors prioritize their teams and consistently communicate their dedication to the institutional missions. The directors recognize that they set an example by their hard work, commitment to the institutions’ missions and the libraries, love of learning, honesty, willingness to embrace change, and confidence in facing their own fears. When they model these behaviors, their employees can, and will, do it, too.

## CONTINUING THE CHANGE PROCESS

Kotter’s eight steps offer a clear framework to help guide change. By leveraging these steps, these two health sciences library directors could quickly identify the necessary people and activities to empower evolution of their libraries to be better positioned to meet the information challenges of the future. While Kotter’s steps are shared here in the context of launching a new vision for two libraries, Kotter’s framework is a useful tool to manage change across many areas in a library, including implementing a new service, restructuring the organization, and managing change following internal assessment or strategic planning. The steps enable employee engagement with the change process, which facilitates ongoing change. Kotter’s steps offer an effective strategy for leading change and empower libraries to engage with campus leadership as true partners supporting the academic, clinical, and research excellence of their universities.
